# Immunological dysregulation of follicular helper T cells, follicular regulatory T cells, and follicular cytotoxic T cells is associated with disease activity and identifies exploratory immune patterns in rheumatoid arthritis

**DOI:** 10.3389/fimmu.2026.1941650

**Published:** 2026-09-04

**Authors:** Yuxin Fan, Baochen Li, Ruihe Wu, Xiaoyang Liu, Xiaoyu Zi, Yuhuan Xie, Chong Gao, Caihong Wang

**Affiliations:** 1 Department of Rheumatology, The Second Hospital of Shanxi Medical University, Taiyuan, Shanxi, China; 2 Shanxi Key Laboratory of Rheumatism Immune Microecology, Taiyuan, Shanxi, China; 3 Shanxi Precision Medical Engineering Research Center for Rheumatology, Taiyuan, Shanxi, China; 4 Department of Pathology, Brigham and Women’s Hospital, Harvard Medical School, Boston, MA, United States

**Keywords:** follicular cytotoxic T cells, follicular helper T cells, follicular regulatory T cells, immunological heterogeneity, rheumatoid arthritis

## Abstract

**Background:**

The onset and progression of rheumatoid arthritis (RA) are linked to autoantibody production driven by dysregulated germinal center (GC) responses. Follicular helper T cells (Tfh), follicular regulatory T cells (Tfr), and follicular cytotoxic T cells (Tfc) are important components of follicular immune regulation. However, the circulating profiles and clinical associations of these three cell subsets in the peripheral blood of RA patients remain underexplored through systematic investigations, and the specific function of Tfc cells in RA remains incompletely understood.

**Methods:**

In the present study, peripheral blood samples were collected from 41 patients with RA and 16 healthy controls (HCs). Modified flow cytometry was employed to detect circulating Tfh, Tfr, and Tfc cells, with the aim of systematically investigating the clinical and immunological implications of these cell subsets in RA.

**Results:**

(1) RA patients exhibited a disrupted Tfh/Tfr ratio in peripheral blood, accompanied by an elevated proportion of Tfc cells, both of which correlated with disease activity and autoantibody levels. (2) The ratio of Tfh/Tfr and the proportion of Tfc cells were significantly increased in patients with high disease activity of RA. (3) The frequency of Tfc cells showed a positive correlation with the frequency of Tfh cells and soluble interleukin-2 receptor (sIL-2R) levels. (4) Based on k-means unsupervised clustering analysis, patients with RA were grouped into three exploratory follicular T-cell-associated immune patterns: Cluster 3 patients displayed the highest disease activity, while Cluster 2 patients presented with the lowest white blood cell count.

**Conclusion:**

The dynamic imbalance of the circulating Tfh/Tfr/Tfc cells may reflect immune dysregulation in RA. The expansion of Tfc cells may be associated with systemic immune activation in RA. Exploratory clustering analysis further identified distinct follicular T-cell-associated immune patterns, providing new insights into RA immunological heterogeneity.

## 1 Introduction

Rheumatoid arthritis (RA) is a chronic systemic autoimmune disease characterized by symmetrical, persistent polyarticular synovitis and erosive joint destruction (1). Excessive production of aberrant autoantibodies, including anti-citrullinated protein antibodies (ACPA) and rheumatoid factor (RF), has been demonstrated to play a pivotal role in the pathogenesis and progression of RA (2, 3). While the exact mechanisms underlying RA remain incompletely elucidated, dysregulated immune tolerance in the peripheral blood and synovium is likely a critical driver of aberrant antibody overproduction (4, 5). Therefore, a better understanding of the immune processes involved in autoantibody production may provide further insight into the immunopathogenesis of RA and potentially relevant immune alterations.

The germinal center (GC) serves as a pivotal niche where B cells undergo clonal expansion, affinity maturation, class-switch recombination, and somatic hypermutation—processes that ultimately yield large quantities of high-affinity antibodies and memory B cells (6, 7). The production of autoantibodies in RA is tightly linked to aberrant activation of GC responses within lymphoid follicles (8). The precise orchestration of this process relies on the dynamic equilibrium between follicular helper T (Tfh) cells and follicular regulatory T (Tfr) cells within the GC. Tfh cells, characterized by high expression of C-X-C chemokine receptor 5 (CXCR5), home to B cell follicles and interact with B cells via the ICOS-ICOSL axis, CD40L-CD40 signaling, and TCR-peptide-MHC II engagement, thereby robustly promoting B cell maturation and the generation of high-affinity antibodies (9, 10). By contrast, Tfr cells arise from FoxP3+ regulatory T (Treg) cell precursors and exert suppressive effects on antibody production through negative regulation of Tfh cells and GC reactions (11, 12). Accumulating evidence indicates that Tfh/Tfr imbalance in RA patients correlates with disease activity and excessive autoantibody production (13, 14), which in turn disrupts immune tolerance (15). However, attributing the overproduction of autoantibodies in RA exclusively to the breakdown of immune tolerance mediated by Tfh/Tfr imbalance appears to be an oversimplification.

Recently, alongside CD4+T cell subsets, a novel CD8+T cell subset has been identified in human tonsils, designated as follicular cytotoxic T cells (Tfc) (16). To date, a consensus definition of its phenotype remains elusive; herein, we adopt the characterization proposed by Kristen M. Valentine, Xuwen Zhai, and colleagues, which defines Tfc cells as CD8+CXCR5+PD-1+T cells (17, 18). Distinct from conventional effector CD8+T cells, Tfc cells exhibit unique phenotypic and functional heterogeneity. On one hand, they retain the cytotoxic potential inherent to CD8+T cells, expressing granzyme and perforin; on the other hand, they co-express CD8, CXCR5, co-stimulatory molecules (such as ICOS, CD40L), and transcription factors (such as Bcl-6), localize to B cell follicles and their adjacent microenvironments, and display a transcriptional profile analogous to that of Tfh cells (19, 20). This distinctive molecular signature equips Tfc cells with the capacity to execute complex immune regulatory functions within lymphoid follicles. Initially, Tfc cells were extensively investigated in the context of infection and tumorigenesis. In chronic infection or tumor microenvironments, Tfc cells are regarded as pre-exhausted or stem-like CD8+T cells that respond favorably to PD-1/PD-L1 immune checkpoint blockade. They sustain long-lasting antiviral or antitumor immune responses through self-renewal and differentiation (19, 21–24). However, their role in autoimmune diseases is more intricate. Studies have demonstrated a significant increase in the frequency of Tfc cells in the peripheral blood of patients with primary Sjögren’s syndrome (pSS) and multiple sclerosis (MS), which positively correlates with the counts of B cells and Tfh cells, suggesting their potential involvement in the helper process of autoreactive B cells (18, 25). Notably, research has shown that in the very early stages of RA onset (even during the preclinical risk period), elevated frequencies of Tfh and Tfc cells are already present in local lymphoid tissues, with a positive correlation to B cell counts. This provides direct histological evidence for the contribution of follicular T cells to the early initiation of RA, though such imbalances have not yet been detected in peripheral blood (26). Therefore, further investigation is needed to clarify the association between circulating Tfc cells, humoral immune abnormalities, and disease activity in RA.

Tfc cells co-localize with Tfh and Tfr cells within lymphoid follicles, and according to existing research, they might interact with each other during the regulation of GC responses. On one side, previous studies suggest that Tfc cells may share certain functional characteristics with Tfh cells. For example, Tfc cells may activate GC B cells by secreting IL-21 and engaging the CD40L/CD40 and ICOSL/ICOS pathways, thereby facilitating the production of high-affinity IgG and IgM by B cells (19, 20). On the other side, Tfc cells have been implicated in the regulation of Tfh cell function, with reports of both positive and negative regulatory effects (19). As negative regulators of GC responses, Tfr cells primarily function to suppress the activity of Tfh cells and GC B cells, preventing excessive autoimmune reactions (11, 12); however, some studies indicate that under certain conditions, Tfr cells may also inhibit Tfc cell proliferation (27). Collectively, Tfc, Tfh, and Tfr cells may participate in a complex regulatory environment within lymphoid follicles, potentially influencing GC B-cell responses and subsequent autoantibody production (28). However, their precise interactions and contributions to autoimmune responses remain to be fully elucidated.

In summary, Tfh, Tfr, and Tfc cells do not exist in isolation. Through intricate intercellular communication and functional crosstalk, they may collectively shape the immune response landscape within RA. However, current studies investigating these three follicular T-cell subsets in the peripheral blood of RA patients remain fragmented and unsystematic, and their integrated relationships with disease activity and immune-related parameters require further clarification. This study intends to compare the expression profiles of Tfh, Tfr, and Tfc cells in the peripheral blood of RA patients, healthy controls, and RA patients with varying disease activity; analyze their correlations with clinical parameters; and perform K-means cluster analysis to identify exploratory follicular T-cell-associated immune patterns among RA patients. Through this approach, we aim to improve the understanding of peripheral follicular T-cell alterations and immune heterogeneity in RA, providing additional insights into the complex immune dysregulation associated with this disease.

## 2 Materials and methods

### 2.1 Study population

In this study, 41 RA patients admitted to the Department of Rheumatology and Immunology of the Second Hospital of Shanxi Medical University between January 2024 and January 2025 were recruited. All patients fulfilled the 2010 RA classification criteria established by the American College of Rheumatology (ACR) and the European Alliance of Associations for Rheumatology (EULAR). Patients with comorbidities such as other autoimmune diseases, malignant neoplasms, severe infections, or severe cardiovascular system disorders, as well as pregnant women or those undergoing low-dose recombinant human IL-2 treatment, were excluded from the study. Moreover, 16 healthy controls were recruited from the Physical Examination Center of our hospital. This study was duly approved by the Ethics Committee of the Second Hospital of Shanxi Medical University for medical research projects, with the approval number (2019) YX No (105). All participants provided written informed consent after being comprehensively informed about the study details.

### 2.2 Clinical and laboratory data collection

We gathered demographic information, clinical data, laboratory data, and current medications of patients with RA. The demographic information encompassed details such as gender, age, height, and weight. Clinical data included the duration of RA, tenderness joint count (TJC), swollen joint count (SJC), etc. Laboratory data involved a comprehensive assessment, including blood routine analysis, liver function tests, kidney function tests, conventional inflammatory biomarker measurements [such as erythrocyte sedimentation rate (ESR) and C-reactive protein (CRP)], and quantification of autoantibody levels (including anti-cyclic citrullinated peptide (Anti-CCP), anti-mutated citrullinated vimentin (Anti-MCV), and RF). The current medications included oral prednisone, disease-modifying antirheumatic drugs (DMARDs), and biological agents. The disease activity of RA patients was evaluated using the Disease Activity Score 28 joint count (DAS28)-ESR system. Based on their DAS28-ESR scores, RA patients were stratified into two groups: Non-High Disease Activity Group (non-HDA) with a score ≤ 5.1, and the High Disease Activity Group (HDA) with a score>5.1.

### 2.3 Flow cytometry measurement of the percentages and absolute counts of peripheral blood, CD4^+^T, CD8^+^T, Tfh, Tfr, and Tfc cell subsets

Peripheral blood samples (2 mL) were collected in heparin-anticoagulated tubes. Circulating Tfh, Tfr, and Tfc cells were assessed by modified flow cytometry. The modification involved refinement of the definition and quantification strategies for follicular T-cell subsets. Representative plots are shown in the Supplementary material: **Supplementary Figure S1**. Experimental details are provided in the “Methods” section of the **Supplementary Material** (**Supplementary Material**: **Supplementary Table 1**).

### 2.4 Detection of cytokine concentrations by cytometric bead array

Serum samples were isolated from 4 mL of peripheral venous blood and stored at −20 °C until analysis. The concentrations of soluble interleukin-2 receptor (sIL-2R), IL-2, interleukin-4 (IL-4), interleukin-6 (IL-6), interleukin-10 (IL-10), interleukin-17 (IL-17), interferon-γ (IFN-γ), and tumor necrosis factor-α (TNF-α) in the serum were measured using a cytometric bead array (CBA) assay based on flow cytometry according to the manufacturer’s instructions. Detailed experimental procedures are described in the “Methods” section of the **Supplementary Material**.

### 2.5 Statistical analysis

All quantitative data were first subjected to normality testing (Shapiro-Wilk test) and homogeneity of variance testing (Levene’s test). For normally distributed data, results were presented as mean ± standard deviation ( $\bar{x}$ ± s) and compared using independent samples t-tests (two groups) or one-way analysis of variance (ANOVA; multiple groups) followed by Bonferroni *post hoc* correction for pairwise comparisons. Bivariate correlations were assessed via Pearson’s correlation analysis. For non-normally distributed data, results were expressed as median and interquartile range [M (Q25, Q75)]; comparisons between two groups were performed using the Mann-Whitney U test, while comparisons across multiple groups employed the Kruskal-Wallis H test followed by Dunn’s *post hoc* test for pairwise comparisons. Bivariate correlations for non-normal data were analyzed via Spearman’s rank correlation analysis. Categorical variables were presented as numbers and percentages and compared using Fisher’s exact test, followed by Bonferroni *post hoc* correction for pairwise comparisons. Considering the large number of correlation tests performed, P values were adjusted using the Benjamini–Hochberg false discovery rate (FDR) method to control for multiple comparisons. A two-tailed P value < 0.05 was considered statistically significant. Missing values with a missing rate < 20% were imputed using R software (version 4.5.1). Data analysis and visualization were conducted using SPSS 25.0, GraphPad Prism 10.0, Origin 2021, and R software (version 4.5.1). Unsupervised K-means clustering analysis and visualization were performed using R software (version 4.5.1).

## 3 Results

### 3.1 Clinical and demographic features

Demographic characteristics, clinical features, and laboratory data of 41 RA patients and 16 healthy controls are summarized in Supplementary material: **Supplementary Table 2**. No significant differences were observed in demographic data between RA patients and HCs.

Demographic characteristics, clinical features, laboratory data, and current medications of 18 non-HDA and 23 HDA RA patients are presented in **Table 1**. No significant differences were noted in demographic data or current medications between the non-HDA and HDA groups.

**Table 1:** The summary of demographic information, clinical data, laboratory data, and current medications in the non-HDA and HDA groups.

Characteristics	Non-HDA (n=18)	HDA (n=23)	P value
Demographic information			
Male, n (%) ^c^	6(33.3%)	10(43.5%)	0.509
Female, n (%) ^c^	12(66.7%)	13(56.5%)	
Age (years) ^a^	59.39 ± 10.04	61.00 ± 12.25	0.654
Weight(kg) ^b^	62.5(60.00-68.50)	62.50(55.00-65.00)	0.357
Height(cm) ^a^	164.17 ± 7.54	162.57 ± 8.39	0.530
Clinical data			
Disease duration (months) ^b^	66 (33–150)	36(5-96)	0.087
TJC ^b^	3(1-9)	18(10-24)	<0.001***
SJC ^b^	1(0-3)	4(1-20)	0.015*
DAS28 ^b^	4.25(3.98-4.93)	6.80(6.30-7.50)	<0.001***
Laboratory data			
WBC (×10^9/L) ^b^	6.31(5.01-8.33)	7.08(5.80-8.16)	0.372
LY (×10^9/L) ^a^	1.44 ± 0.52	1.39 ± 0.54	0.765
Hb (g/L) ^b^	129.5(115.0-134.5)	116.0(105.0-125.0)	0.053
PLT (×10^9/L) ^a^	247.22 ± 56.82	284.65 ± 89.40	0.130
ESR (mm/h) ^b^	19.50(7.00-65.00)	70.00(48.00-103.00)	0.004**
CRP (mg/L) ^b^	16.15(7.46-55.58)	41.51(16.10-68.79)	0.338
Liver function			
ALT (U/L) ^b^	16.10(11.98-36.13)	19.70(15.20-43.30)	0.454
AST (U/L) ^b^	20.00(15.85-23.73)	22.20(15.60-30.90)	0.400
Renal function			
BUN (mmol/L) ^a^	6.11 ± 1.95	6.94 ± 2.49	0.251
Cr (μmol/L) ^a^	60.93 ± 14.31	53.44 ± 11.79	0.074
Autoantibody			
Anti-CCP (U/mL) ^b^	368.01(44.42-550.00)	550.00(201.12-550.00)	0.141
Anti-MCV (U/mL) ^b^	188.45(3.00-1100.00)	769.20(140.00-1100.00)	0.250
RF-IgA (U/mL) ^b^	48.55(3.00-269.23)	89.30(3.00-232.10)	0.790
RF-IgG (U/mL) ^b^	56.20(3.00-156.28)	53.40(3.00-176.60)	0.979
RF-IgM (U/mL) ^b^	189.10(18.38-309.65)	206.90(38.00-350.00)	0.592
Current medications			
Oral prednisone (≤10 mg/day), n (%) ^c^	13(72.2%)	12(52.2%)	0.192
DMARDs, n (%) ^d^	17(94.4%)	19(82.6%)	0.363
Biological agent, n (%) ^d^	3(16.7%)	3(13.0%)	1.000

HC, healthy control; RA, rheumatoid arthritis; TJC, tenderness joint count; SJC, swollen joint count; DAS 28, disease activity score 28; WBC, white blood cell; LY, lymphocyte; Hb, hemoglobin; PLT, platelet; ESR, erythrocyte sedimentation rate; CRP, C-reactive protein; ALT, alanine transaminase; AST, aspartic transaminase; BUN, blood urea nitrogen; Cr, creatinine; Anti-CCP, anti-cyclic citrullinated peptide; Anti-MCV, anti-mutated citrullinated vimentin; RF, rheumatoid factor.

^a^Results were expressed as the mean ± SD and were analyzed by independent-samples t-tests.

^b^Results were expressed as the median (Q1-Q3) and were analyzed by Mann–Whitney U test.

^c^Categorical variables were described as rates and percentages and were assessed using the chi-square tests.

^d^Categorical variables were described as rates and percentages and were assessed using the Fisher’s exact tests.

Bold P values indicate statistical significance (P < 0.05). *P<0.05, **P<0.01, ***P<0.001.

### 3.2 Dysregulated Tfh/Tfr ratio and Tfc% expansion in rheumatoid arthritis

**Figure 1:**
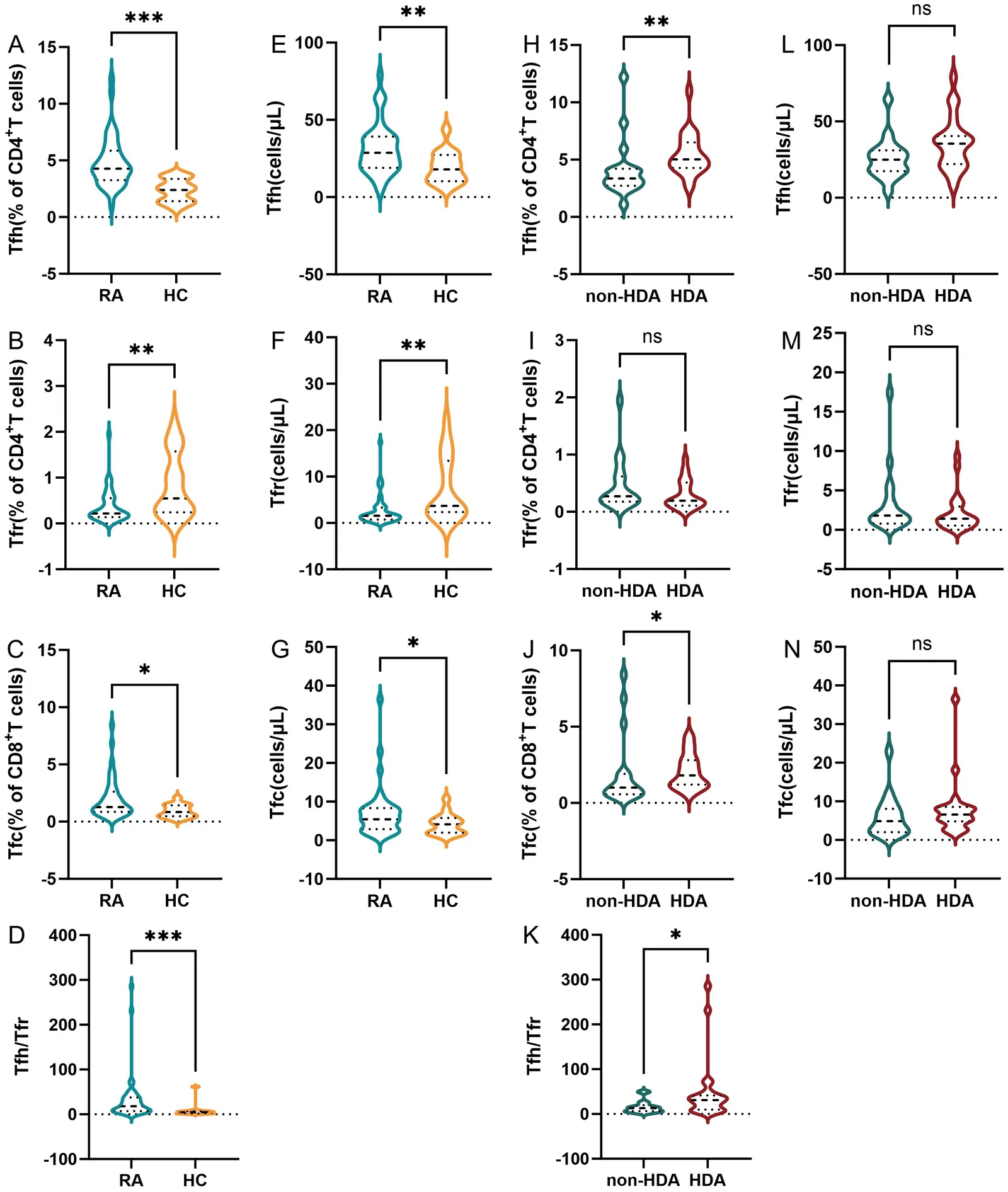
Comparisons in the expression of Tfh, Tfr, and Tfc cells between patients with RA and HCs, and comparisons in the expression of Tfh, Tfr, and Tfc cells between patients in the non-HDA group and those in the HDA group. **(A–D)** Comparison of Tfh percentage, Tfr percentage, Tfc percentage, and Tfh/Tfr ratio between RA patients and HCs. **(E–G)** Comparison of absolute counts of Tfh, Tfr, and Tfc cells between RA patients and HCs. **(H–K)** Comparison of Tfh percentage, Tfr percentage, Tfc percentage, and Tfh/Tfr ratio between non-HDA and HDA groups. **(L–N)** Comparison of absolute counts of Tfh, Tfr, and Tfc cells between non-HDA and HDA groups. Tfh and Tfr frequencies are presented as percentages of CD4^+^T cells, whereas Tfc frequencies are presented as percentages of CD8^+^T cells. Absolute cell counts are expressed as cells/μL. Data in panel L were normally distributed and analyzed using an independent-samples *t*-test; data in panels **(A–K, M, N)** were non-normally distributed and analyzed using the Mann–Whitney U test. HCs, healthy controls. non-HDA, Non-High Disease Activity Group. HDA, High Disease Activity group. Tfh, follicular helper T cells. Tfr, follicular regulatory T cells. Tfc, follicular cytotoxic T cells. ns, not significant. *P<0.05, **P<0.01, ***P<0.001.

The frequencies of Tfh and Tfr cells among CD4^+^T cells, and Tfc cells among CD8^+^T cells in peripheral blood were compared between the RA group and the HC group. The results indicated that, compared with the HC group, Tfh% [4.27 (3.26, 5.86) % vs. 2.40 (1.40, 3.37) %, P < 0.001], Tfc% [1.27 (0.85, 2.63) % vs. 0.84 (0.48, 1.42) %, P = 0.011], and the ratio of Tfh/Tfr [18.10 (7.13, 37.58) vs. 4.69 (1.83, 6.88), P < 0.001] were all elevated in the peripheral blood of the RA group (**Figures 1A, C, D**). Conversely, Tfr% [0.22 (0.14, 0.55) % vs. 0.55 (0.24, 1.57) %, P = 0.008] was decreased in the RA group compared to the HC group (**Figure 1B**).

Furthermore, the absolute counts of Tfh, Tfr, and Tfc cells in peripheral blood were compared between the two groups. The results demonstrated that, relative to the HC group, the absolute count of Tfh cells [28.75 (18.88, 39.13) cells/µL vs. 17.89 (10.31, 27.32) cells/µL, P = 0.006], Tfc cells [5.45 (2.86, 8.35) cells/µL vs. 4.14 (2.00, 5.76) cells/µL, P = 0.030] in the peripheral blood of the RA group was higher (**Figures 1E, G**). Conversely, the absolute count of Tfr cells [1.55 (0.72, 3.30) cells/µL vs. 3.70 (2.40, 13.40) cells/µL, P = 0.004] was decreased in the RA group compared to the HC group (**Figure 1F**).

### 3.3 Tfh/Tfr imbalance and Tfc expansion characterize high disease activity in RA

We compared the frequencies of Tfh and Tfr cells among CD4^+^T cells, and Tfc cells among CD8^+^T cells in peripheral blood between RA patients in the non-HDA group and those in the HDA group. Compared with the non-HDA group, the HDA group exhibited significantly higher peripheral blood Tfh% [5.02 (4.27, 6.51) % vs. 3.37 (2.72, 4.21) %, P = 0.003], Tfc% [1.81 (1.21, 2.80) % vs. 1.00 (0.57, 1.91) %, P = 0.037], and Tfh/Tfr ratio [30.92 (9.67, 41.33) vs. 13.13 (6.30, 19.60), P = 0.016] (**Figures 1H, J, K**). No statistically significant difference was observed in Tfr% between the two groups (**Figure 1I**).

We further compared the absolute counts of these cell subsets between the non-HDA and HDA groups. No statistically significant differences were noted in the absolute counts of Tfh, Tfr, or Tfc cells between the two groups (**Figures 1L, M, N**).

### 3.4 Associations between follicular T-cell subsets and clinical parameters in patients with RA

**Figure 2:**
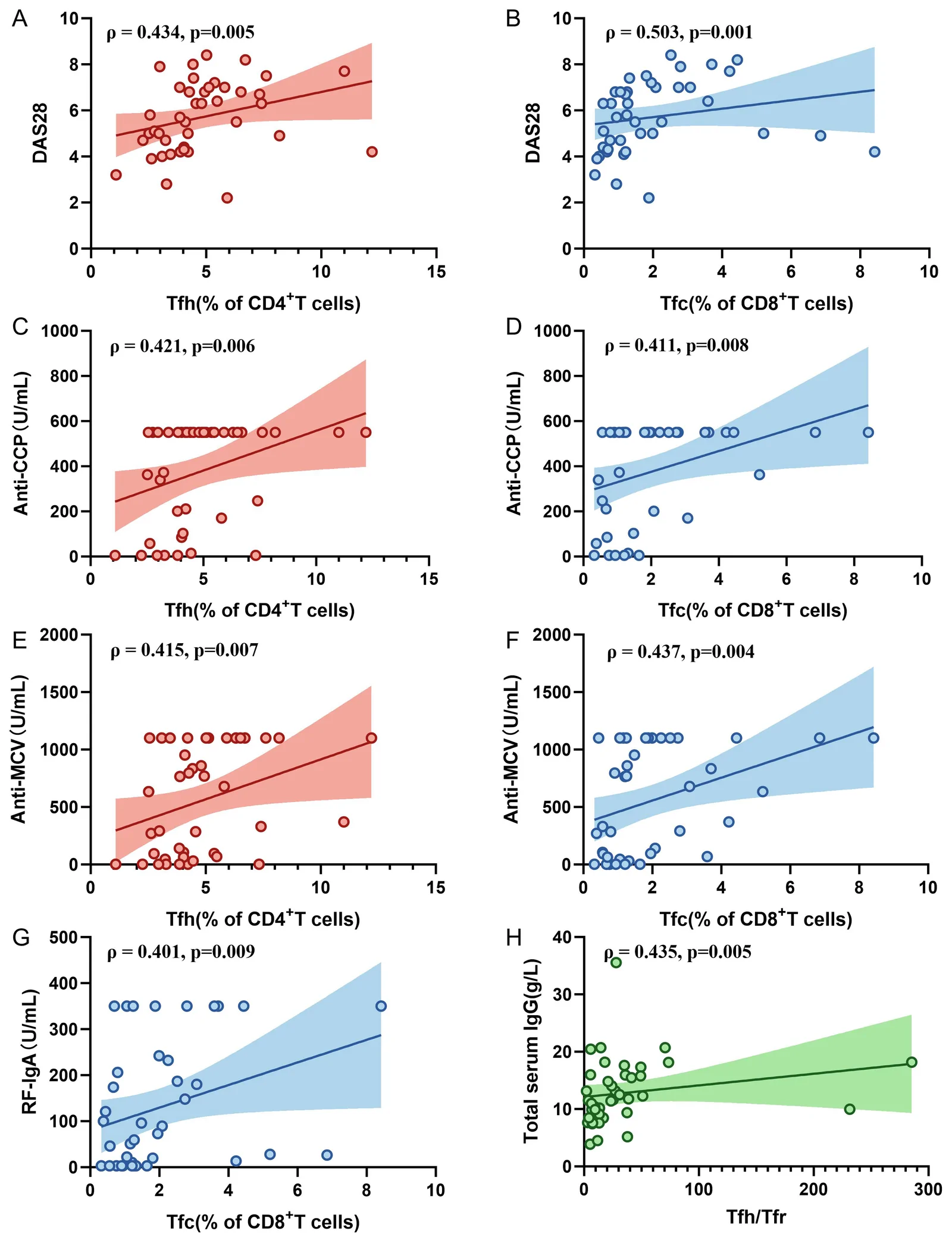
Representative correlations between circulating follicular T-cell subsets and clinical parameters in patients with RA. Representative scatter plots illustrating significant correlations between circulating follicular T-cell subsets and clinical indicators after Benjamini–Hochberg false discovery rate (FDR) correction. Spearman’s rank correlation coefficients (ρ) and nominal P values are shown in each panel. Simple linear regression lines with 95% confidence intervals are displayed for visualization of the correlation trends. **(A)** Correlation between the percentage of Tfh cells among CD4^+^T cells and DAS28. **(B)** Correlation between the percentage of Tfc cells among CD8^+^T cells and DAS28. **(C)** Correlation between the percentage of Tfh cells among CD4^+^T cells and Anti-CCP levels. **(D)** Correlation between the percentage of Tfc cells among CD8^+^T cells and Anti-CCP levels. **(E)** Correlation between the percentage of Tfh cells among CD4^+^T cells and Anti-MCV levels. **(F)** Correlation between the percentage of Tfc cells among CD8^+^T cells and Anti-MCV levels. **(G)** Correlation between the percentage of Tfc cells among CD8^+^T cells and RF-IgA levels. **(H)** Correlation between Tfh/Tfr ratio and total serum IgG concentration (IgG) levels. Tfh and Tfr frequencies are presented as percentages of CD4^+^T cells, whereas Tfc frequencies are presented as percentages of CD8^+^T cells. Tfh: follicular helper T cells. Tfr, follicular regulatory T cells. Tfc, follicular cytotoxic T cells. DAS28, disease activity score 28. IgG, total serum immunoglobulin G.

We next investigated the associations between circulating follicular T-cell subsets and clinical parameters in patients with RA. After Benjamini–Hochberg false discovery rate (FDR) correction for multiple comparisons, Tfh% was positively associated with DAS28 score, Anti-CCP and Anti-MCV levels, while Tfc% showed positive associations with DAS28 score, RF-IgA, Anti-CCP and Anti-MCV levels. In addition, Tfh/Tfr ratio showed a positive association with total serum IgG concentration (IgG) (Supplementary material: **Supplementary Figure S2**). FDR-significant associations were further visualized using scatter plots with fitted regression lines for visualization (**Figure 2**). Detailed correlation coefficients (ρ), 95% confidence intervals (CIs), nominal P values, and FDR-adjusted P values are provided in **Supplementary Table 3**.

### 3.5 Correlation between Tfh/Tfr/Tfc cells and cytokine levels in RA patients

**Figure 3:**
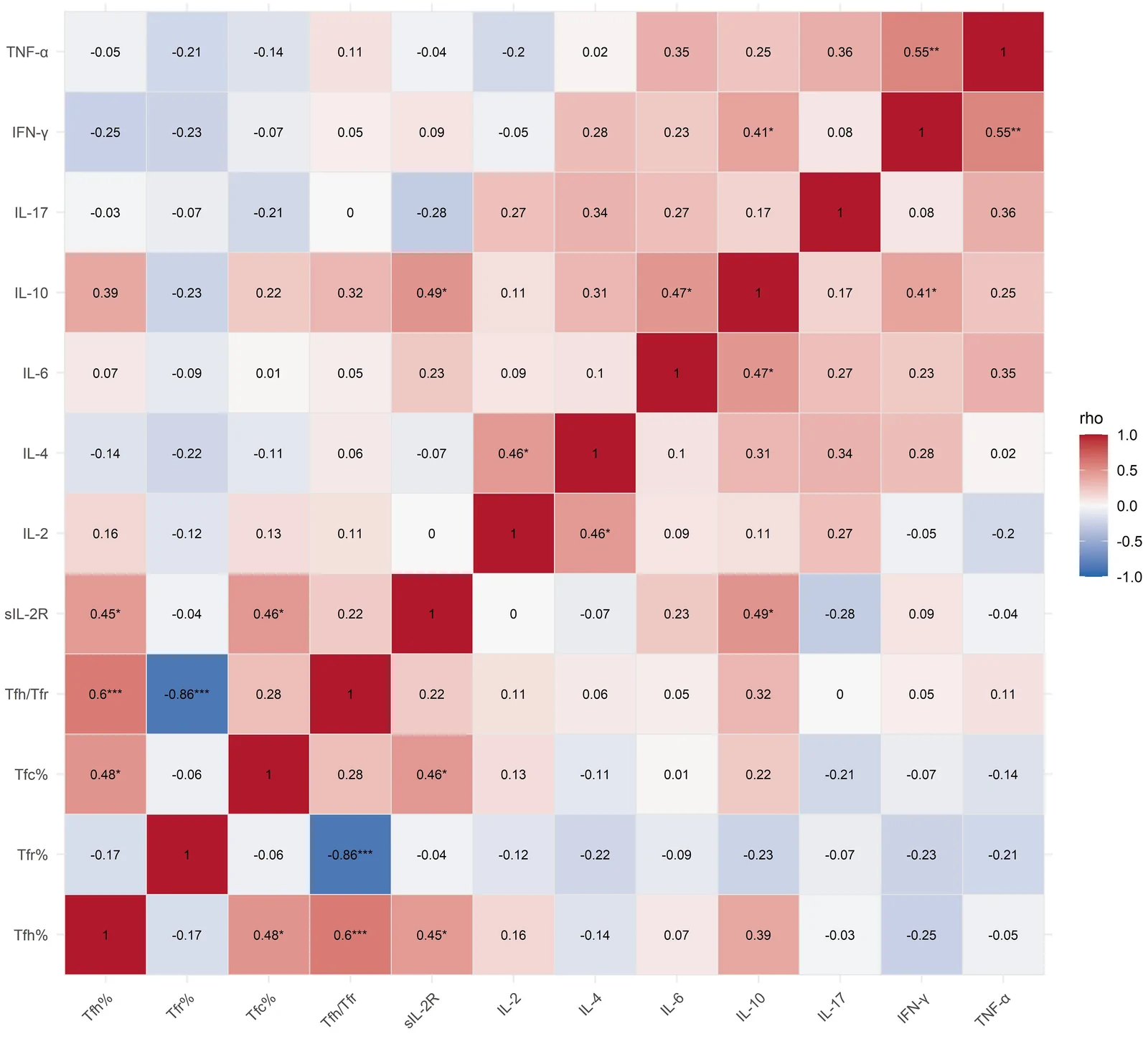
Correlation analysis between circulating follicular T-cell subsets and serum cytokine levels in patients with RA. Spearman’s rank correlation analysis was performed to evaluate the associations between circulating follicular T-cell subsets and serum cytokine concentrations in patients with rheumatoid arthritis. Multiple comparisons were corrected using the Benjamini–Hochberg false discovery rate (FDR) method. Asterisks indicate FDR-adjusted statistical significance (*P<0.05, **P<0.01, ***P<0.001).

We further explored the associations between circulating follicular T-cell subsets and serum cytokine levels in patients with RA. After FDR correction, the results showed that Tfc% was positively correlated with Tfh% and sIL-2R levels (**Figure 3**).

### 3.6 Exploratory immune pattern identification based on Tfh and Tfc profiles

**Figure 4:**
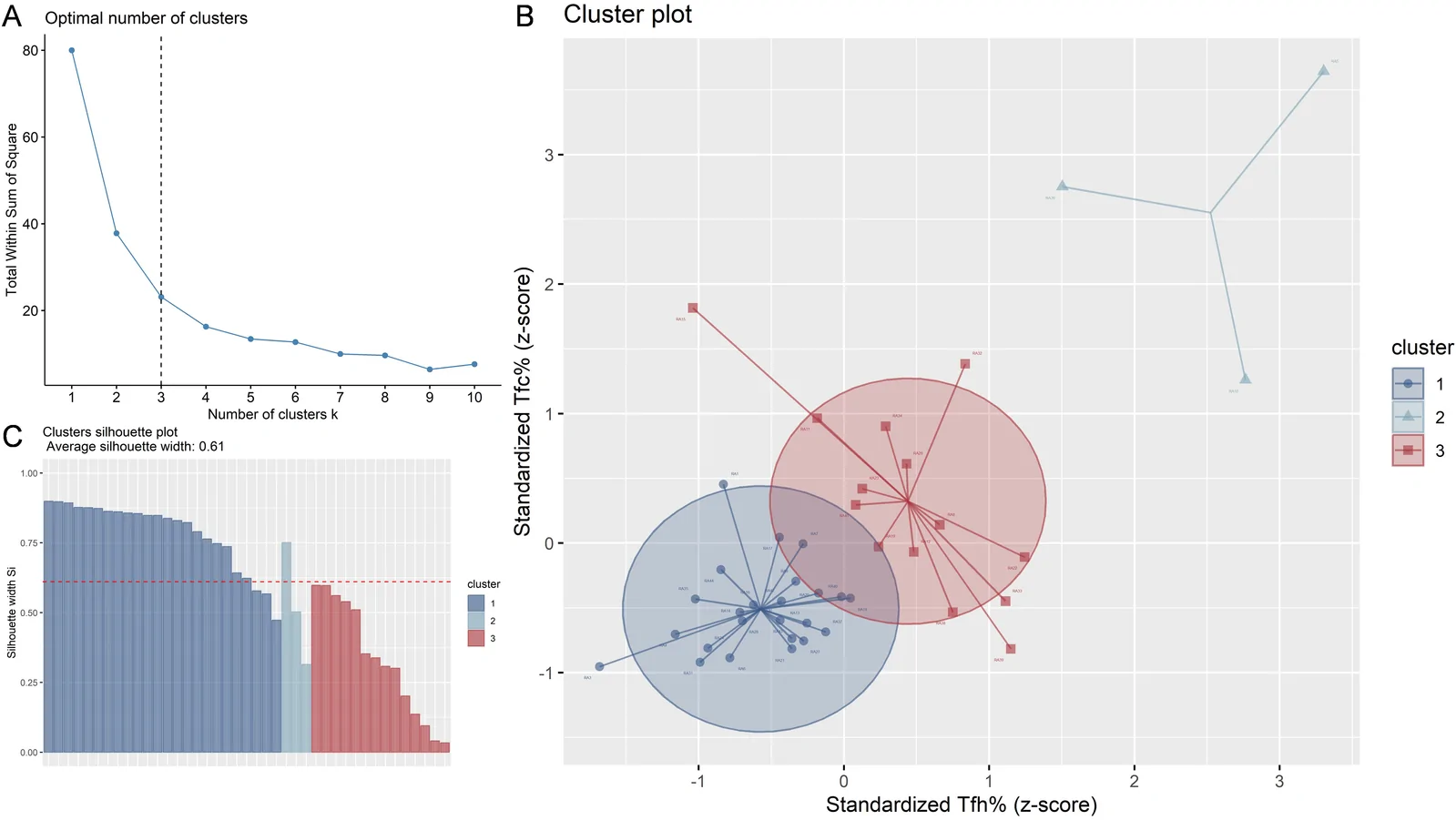
Exploratory clustering analysis based on the K-means algorithm (n = 41). **(A)** Elbow plot showing the within-cluster sum of squares (WSS) across different numbers of clusters. The levelling-off point supported the selection of three clusters (K = 3). **(B)** Cluster plot generated by K-means clustering (K = 3). Each point represents an individual RA patient, and colors indicate different clusters. **(C)** The average silhouette plot. The dashed lines in the image indicate the average silhouette width of the three clusters.

Cluster analysis was performed using Tfh% and Tfc%. Before clustering, variables were standardized using z-score transformation to eliminate the influence of different measurement scales. To assess the clusterability of the dataset, the Hopkins statistic was calculated. It was observed that the Hopkins statistic value of the dataset was 0.663239, indicating a moderate clustering tendency and supporting the feasibility of partitioning the dataset into distinct clusters. The optimal number of clusters (K = 3) was determined via the Elbow method (**Figure 4A**). Based on z-score standardized Tfh% and Tfc% values, K-means unsupervised clustering analysis identified three exploratory immune patterns among RA patients (**Figure 4B**):

Cluster 1 (n=24): Characterized by low Tfh% and low Tfc%;

Cluster 2 (n=3, 7.3% of total): Characterized by high Tfh% and high Tfc%;

Cluster 3 (n=14): Characterized by intermediate Tfh% and intermediate Tfc% (**Supplementary Material**: **Supplementary Table 4**).

The clustering quality and robustness were evaluated by the silhouette coefficient index, hierarchical clustering, bootstrap stability analysis, and consensus clustering. The average silhouette width of the three clusters was 0.610804, indicating well-separated clusters (**Figure 4C**). Hierarchical clustering generated a comparable three-branch structure, supporting the presence of three major immunological patterns. Bootstrap resampling demonstrated acceptable stability of the identified clusters, with Jaccard similarity values above 0.80 for all three clusters. Furthermore, consensus clustering supported K = 3 as an appropriate clustering solution (**Supplementary Material**: **Supplementary Figure S3**).

### 3.7 Comparison of clinical characteristics across exploratory immune patterns in RA

**Supplementary Material**: **Supplementary Table 5** summarizes the demographic and clinical features of the study cohort, which were classified into three clusters based on the K-means clustering of Tfh% and Tfc%. No significant differences were observed in height, weight, or disease duration among the three clusters. Cluster 1 RA patients were predominantly middle-aged (55.63 ± 11.28 years, P = 0.004) and female (83.3%, P = 0.002), with the lowest Anti-MCV [205.80(16.85-827.85) U/mL, P = 0.034]. Cluster 2 patients had the lowest white blood cell (WBC) count (4.20 ± 1.99×10^9/L, P = 0.049). Cluster 3 patients had the highest tenderness joint count (TJC) [20 (9–24), P = 0.040] and disease activity score (DAS28) [6.90(6.30-7.50), P = 0.017].

**Figure 5:**
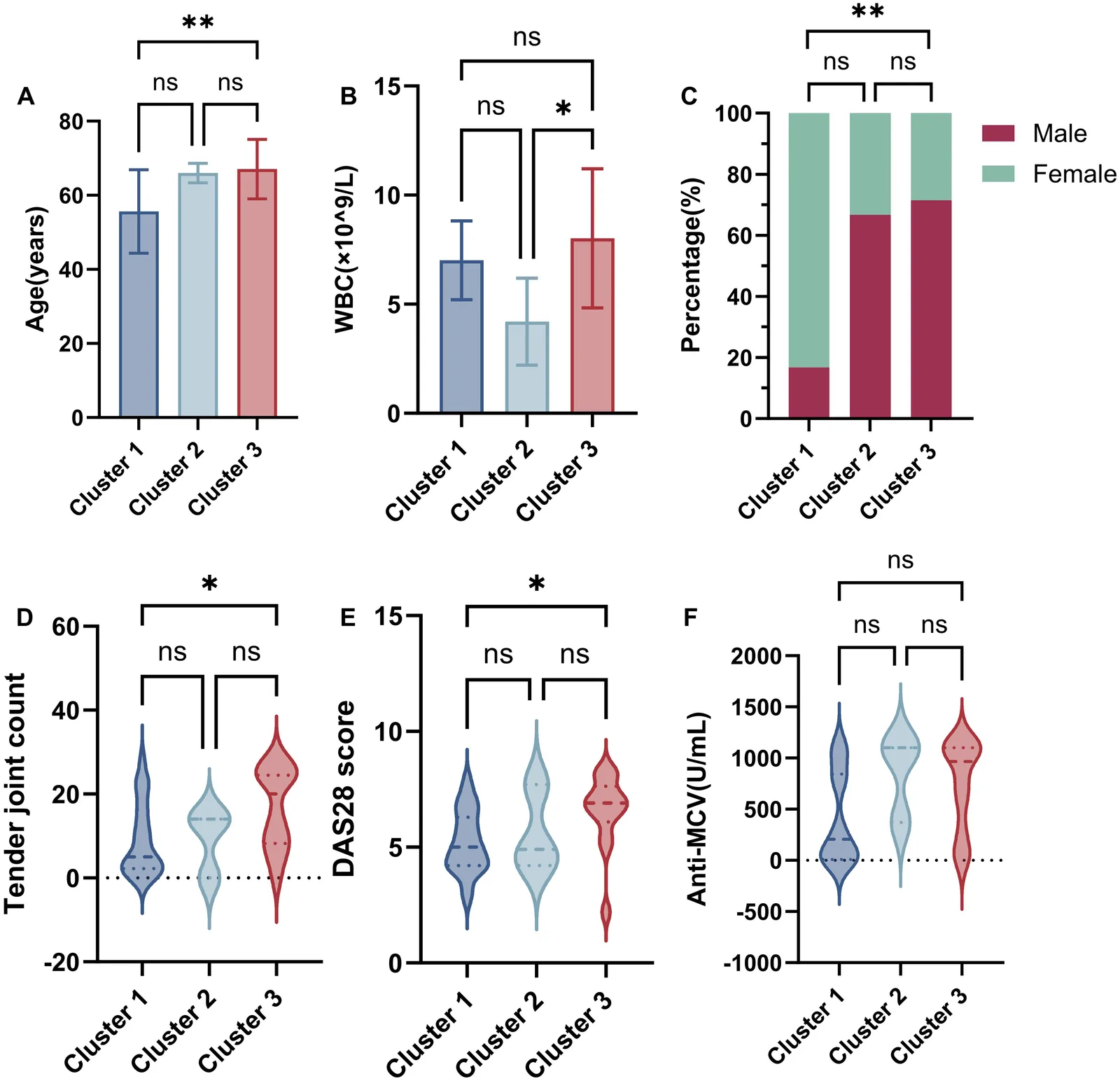
Three exploratory clusters of patients with RA showed statistically significant demographic and clinical characteristics after K-means clustering based on standardized Tfh% and Tfc%. **(A)** Age distribution among clusters. Data were normally distributed and showed homogeneity of variance. Continuous variables were expressed as mean ± SD and compared using ANOVA, followed by Bonferroni *post hoc* correction for pairwise comparisons. **(B)** WBC counts among clusters. Data were normally distributed and showed homogeneity of variance. Continuous variables were expressed as mean ± SD and compared using ANOVA, followed by Bonferroni *post hoc* correction for pairwise comparisons. **(C)** Sex distribution among clusters. Categorical variables were presented as numbers and percentages and compared using Fisher’s exact test, followed by Bonferroni *post hoc* correction for pairwise comparisons. **(D)** TJC among clusters. Data were non-normally distributed. Results were expressed as the median (Q1-Q3) and were analyzed by the Kruskal-Wallis H test, followed by Dunn’s *post hoc* test for pairwise comparisons. **(E)** DAS28 among clusters. Data were non-normally distributed. Results were expressed as the median (Q1-Q3) and were analyzed by the Kruskal-Wallis H test, followed by Dunn’s *post hoc* test for pairwise comparisons. **(F)** Anti-MCV levels among clusters. Data were non-normally distributed. Results were expressed as the median (Q1–Q3) and were analyzed by the Kruskal-Wallis H test, followed by Dunn’s *post hoc* test for pairwise comparisons. WBC, white blood cell; TJC, tenderness joint count; DAS 28, disease activity score 28; Anti-MCV, anti-mutated citrullinated vimentin. ns, not significant. (*P<0.05, **P<0.01).

Pairwise comparisons showed that compared to Cluster 1, patients in Cluster 3 were older (67.07 ± 8.01 years vs. 55.63 ± 11.28 years, P = 0.005), had a higher proportion of males (71.4% vs. 16.7%, P = 0.003), a higher TJC [20 (9–24) vs. 5 (3–16), P = 0.037], and a higher DAS28 [6.90(6.30-7.50) vs. 5.00(4.20-6.30), P = 0.014], while there were no significant statistical differences between cluster 3 and cluster 2 for these variables. Patients in Cluster 3 had a higher white blood cell count (8.02 ± 3.19×10^9/L vs. 4.20 ± 1.99×10^9/L, P = 0.048) compared to those in Cluster 2, while there were no significant statistical differences between Cluster 3 and Cluster 1 for these variables (**Figure 5**).

### 3.8 Cytokine profile comparison across exploratory immune patterns in RA

**Figure 6:**
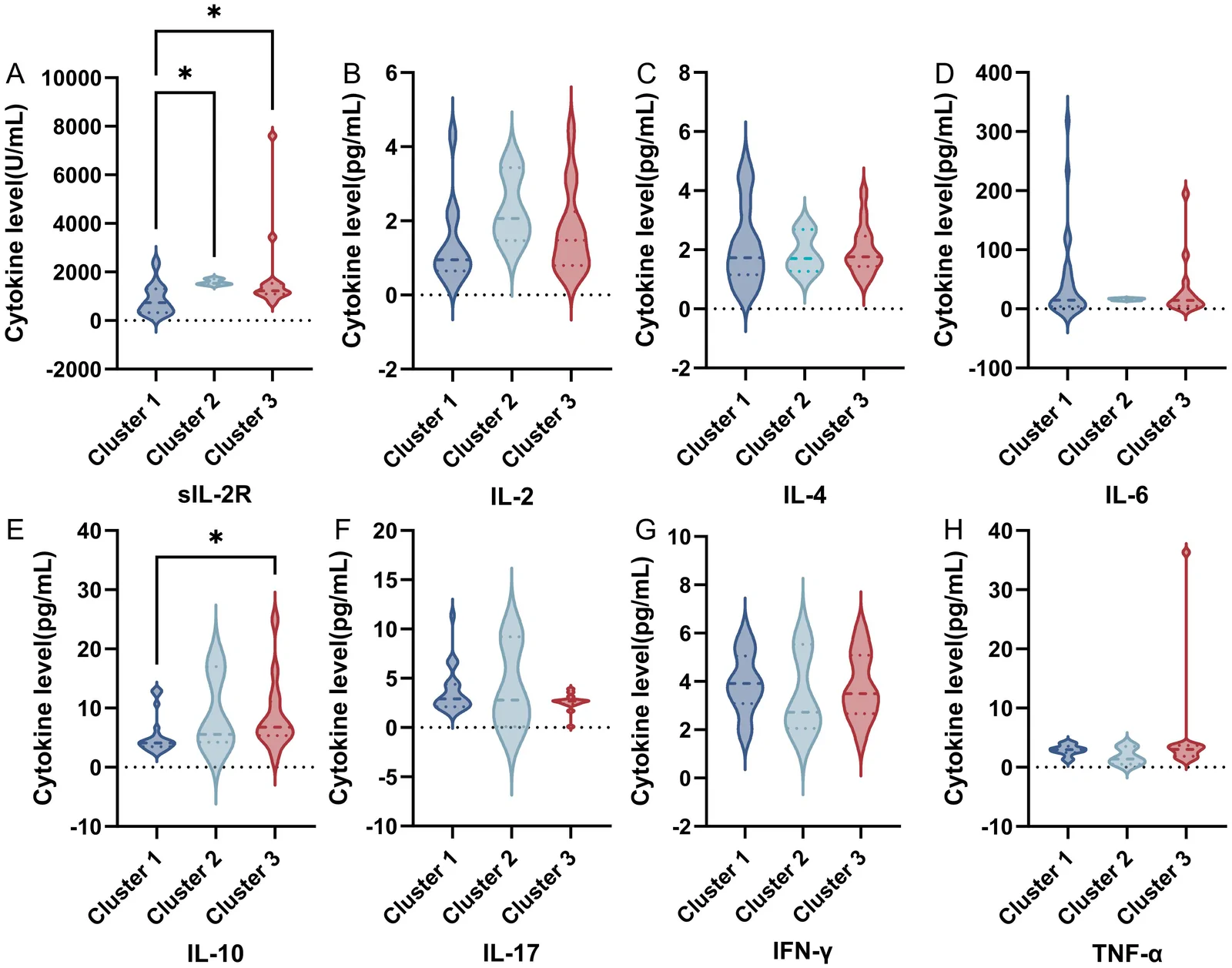
Comparison of cytokines among three exploratory clusters of patients with RA after K-means clustering based on standardized Tfh% and Tfc%. **(A)** Soluble interleukin-2 receptor (sIL-2R) levels among clusters. Data were non-normally distributed and expressed as median (Q1–Q3). Differences among clusters were analyzed using the Kruskal–Wallis H test followed by Dunn’s *post hoc* test with multiple comparison adjustment. **(B)** IL-2 levels among clusters. Data were non-normally distributed and analyzed using the Kruskal–Wallis H test followed by Dunn’s *post hoc* test with multiple comparison adjustment. **(C)** IL-4 levels among clusters. Data were non-normally distributed and analyzed using the Kruskal–Wallis H test followed by Dunn’s *post hoc* test with multiple comparison adjustment. **(D)** IL-6 levels among clusters. Data were non-normally distributed and analyzed using the Kruskal–Wallis H test followed by Dunn’s *post hoc* test with multiple comparison adjustment. **(E)** IL-10 levels among clusters. Data were non-normally distributed and analyzed using the Kruskal–Wallis H test followed by Dunn’s *post hoc* test with multiple comparison adjustment. **(F)** IL-17 levels among clusters. Data were non-normally distributed and analyzed using the Kruskal–Wallis H test followed by Dunn’s *post hoc* test with multiple comparison adjustment. **(G)** IFN-γ levels among clusters. Data were normally distributed with homogeneity of variance. Values were expressed as mean ± SD and analyzed using one-way ANOVA followed by Bonferroni *post hoc* correction for pairwise comparisons. **(H)** TNF-α levels among clusters. Data were non-normally distributed and analyzed using the Kruskal–Wallis H test followed by Dunn’s *post hoc* test with multiple comparison adjustment. sIL-2R, soluble interleukin-2 receptor; IL, interleukin; IFN-γ, interferon gamma; TNF-α, tumor necrosis factor alpha. (*P<0.05).

**Figure 6** illustrates the distinct cytokine profiles among the three clusters. The sIL-2R level in Cluster 1 RA patients was the lowest [733.61 (324.10-1304.43) U/mL, P = 0.005]; in contrast, Cluster 3 presented the highest IL-10 level [6.78 (5.35-11.09) pg/mL, P = 0.026] among all clusters. Pairwise comparisons revealed that patients in Cluster 3 had higher sIL-2R [1220.89 (1093.60-1535.02) U/mL vs. 733.61 (324.10-1304.43) U/mL, P = 0.029] and IL-10 [6.78 (5.35-11.09) pg/mL vs. 4.11 (3.47-5.38) pg/mL, P = 0.034] levels compared to those in Cluster 1; patients in Cluster 2 also had a higher sIL-2R level [1533.17 (1506.88-1626.02) U/mL vs. 733.61 (324.10-1304.43) U/mL, P = 0.039] compared to those in Cluster 1(**Figure 6**).

## 4 Discussion

Our study reveals the following key characteristics of RA patients: (i) The peripheral blood of RA patients exhibits dysregulated follicular T cell profiles, marked by Tfh/Tfr imbalance and Tfc cell enrichment, which correlates with disease activity and excessive autoantibody production; (ii) RA patients with high disease activity display significantly elevated Tfh/Tfr ratios and Tfc frequency, suggesting their potential association with disease activity-related immune alterations; (iii) Tfc cell expansion is not only positively associated with sIL-2R, a key activation mediator in the immunoinflammatory environment, but also likely have a potential synergistic effect with Tfh cells; (iv) Through exploratory K-means clustering analysis based on standardized Tfh/Tfc profiles, RA patients were grouped into three immune patterns. Specifically, Cluster 3 (characterized by moderate Tfh/moderate Tfc) was associated with the highest disease activity. In contrast, Cluster 2 (high Tfh/high Tfc) exhibited the lowest white blood cell count. These findings provide novel insights into follicular T-cell-associated immune heterogeneity in RA. However, whether these immune patterns have clinical utility requires validation in larger, independent cohorts.

T lymphocytes are primarily classified into CD4^+^ helper T cells and CD8^+^ cytotoxic T cells. Previous studies have established that aberrations in the function and quantity of the former play a pivotal role in driving autoantibody production (3). Recently, research both domestically and internationally has increasingly focused on a pair of functionally opposing CD4^+^T cell subsets localized within and adjacent to GC in secondary lymphoid organs, namely Tfh and Tfr cells (29, 30). Existing evidence suggests that circulating Tfr and Tfh cells may originate from GCs and share similar phenotypes and functions with their GC-resident counterparts (31–34). Given the difficulty of obtaining human organ tissues for clinical research, this study used flow cytometry to characterize the changes in peripheral blood Tfh and Tfr cell subsets. Analysis of circulating Tfh and Tfr cell profiles revealed that, compared to healthy controls, RA patients exhibited an elevated Tfh% and a reduced Tfr% in peripheral blood, resulting in a disrupted Tfh/Tfr ratio, which is consistent with findings from prior studies (13, 14, 35). This observation indicates that RA immune dysregulation involves Tfh overactivation and expansion alongside relative Tfr deficiency. The increased Tfh/Tfr ratio reflects disruption of follicular immune homeostasis and was associated with autoantibody levels. Further correlation analysis showed that Tfh% positively correlated with RA disease activity and abnormal autoantibody levels, supporting the involvement of Tfh-related immune alterations in autoantibody production and RA pathogenesis. However, whether these changes directly contribute to autoantibody production requires further functional investigation.

Beyond CD4^+^T helper cells, a novel subset of CD8^+^T cells, termed Tfc cells, has been recently identified (19, 20). Prior studies have demonstrated an increased frequency of Tfc cells in local lymphoid tissues during the very early phase of clinical RA onset, even during the pre-clinical risk period. However, this immunological imbalance is not mirrored in peripheral blood (26). Building upon this existing scientific framework, our study compared Tfc cell expression in the peripheral blood of patients with clinically diagnosed RA (36) and healthy controls. Results revealed that Tfc% was significantly elevated in RA patients, with the magnitude of this increase correlating positively with disease activity and autoantibody levels. This finding supports that Tfc cell alterations are also associated with autoantibody production and RA-related immune abnormalities and suggests that as RA becomes clinically established, alterations in Tfc cells may extend beyond local immunopathological sites and become detectable in the peripheral circulation, suggesting their potential involvement in systemic immune dysregulation.

Interestingly, when patients were stratified according to disease activity, RA patients with high disease activity exhibited higher Tfh/Tfr ratios and increased Tfc frequencies compared with those with non-high disease activity. These findings suggest that altered circulating follicular T-cell profiles are associated with disease activity status in RA. Notably, although Tfr cells showed no statistical difference between the two groups, the increased Tfh/Tfr ratio indicates a shift in the relative balance between follicular helper and regulatory T-cell populations. This highlights that dysregulation of the immune regulatory network may play a more critical role in disease exacerbation than changes in individual cell populations.

While prior studies have predominantly focused on individual follicular T cell subsets in autoimmune diseases in isolation (10, 12), immune regulation involves interactions among multiple immune cell populations. Thus, our simultaneous profiling of these three cell populations in RA peripheral blood provides additional insights into the heterogeneity of circulating follicular T-cell subsets in RA. Existing literature suggests that Tfc cells, beyond their direct B cell helper function, may indirectly modulate Tfh activity to influence autoantibody production (19). However, the functional relationship between these populations in autoimmune diseases remains unclear. Our data demonstrate a positive correlation between Tfc and Tfh cells in RA. Tfh and Tfc cells share several transcriptional regulators, including Bcl6 and Tcf1, according to previous experimental studies (20). This finding in our cohort is consistent with the possibility that Tfc and Tfh cells share partially overlapping regulatory pathways and have a potential synergistic effect, although the underlying mechanisms require further investigation. Notably, prior work in glioma patients showed that Tfr cells potently suppress CD8^+^ T cell proliferation/cytotoxicity and exert even stronger inhibition on Tfc cells (27). However, our correlation analysis revealed no significant association between Tfc and Tfr cells in RA. This finding suggests that the relationship between these populations may be complex or not fully reflected by peripheral blood measurements. Collectively, our findings support a potential association among circulating Tfh, Tfr, and Tfc cell alterations in RA and provide a framework for future studies investigating their functional interactions. Further experimental validation is required to determine whether these follicular T-cell subsets directly regulate each other or contribute to RA pathogenesis.

Our findings further highlight the association between circulating Tfc cells and immune activation-related features in RA. To explore potential relationships between Tfc cells and immune activation markers, we analyzed correlations between Tfc frequency and serum cytokine levels. The results of this study revealed a significant positive correlation between the frequency of Tfc cells and the levels of sIL-2R. sIL-2R, also referred to as soluble CD25(sCD25), is a component of the IL-2Rα chain and is widely recognized as a key serological marker of T cell activation, entering the circulation following immune activation (37–39). Our finding suggests that increased circulating Tfc frequency is associated with immune activation status in RA. Previous studies have shown that activated T cells may contribute to the increased serum sIL-2R levels by expressing high levels of membrane-bound IL-2Rα, which is subsequently cleaved by proteases and released into the circulation (40, 41). Notably, in RA, elevated sIL-2R is not only associated with disease activity and poor prognosis (42) but also its dynamic reduction correlates with clinical improvement (43). Collectively, the positive correlation between Tfc cells and sIL-2R suggests a potential relationship between circulating Tfc cells and systemic immune activation in RA. However, whether Tfc cells are directly involved in regulating sIL-2R levels or merely reflect an activated immune state remains unclear. Further functional and longitudinal studies are required to elucidate the biological mechanisms underlying this association.

The high heterogeneity of RA constitutes one of the primary challenges in clinical diagnosis and management. Classification relying solely on conventional clinical symptoms, serological antibodies, or a single cell subset fails to fully capture its intricate immunopathological underpinnings. In this study, we attempted to perform an exploratory immune pattern analysis based on the percentages of Tfh% and Tfc% via unsupervised K-means clustering analysis. Three exploratory Tfh/Tfc-associated immune patterns were identified within our cohort, showing heterogeneous clinical characteristics. Patients in Cluster 3, who had moderate levels of Tfh and Tfc, exhibited the highest DAS28, whereas patients in Cluster 2, despite having the highest Tfh and Tfc proportions, did not display the highest disease activity. This observation suggests that disease activity may not be determined solely by the abundance of individual follicular T cell subsets but may also reflect the balance and interactions among different immune components. Although correlation analysis showed that circulating Tfh/Tfc related parameters showed significant associations with disease activity-related variables, our exploratory clustering analysis further indicated that the relationship between follicular T cell profiles and clinical severity was not simply linear. One possible explanation is that patients with intermediate Tfh and Tfc frequencies may represent a distinct immune regulatory state associated with increased inflammatory activity. However, this interpretation remains hypothetical and requires further investigation. Notably, these exploratory immune patterns also showed potential differences in humoral immune characteristics. Anti-MCV levels showed an overall difference across clusters, with a trend towards higher levels in Cluster 2 and Cluster 3 compared with Cluster 1. Although *post hoc* pairwise comparisons after correction did not reveal significant differences, this finding may suggest a potential association between distinct circulating follicular T-cell profiles and humoral immune responses.

Furthermore, Cluster 2 displayed relatively lower white blood cell counts. However, this cluster consisted of only three patients, and the biological significance of this observation remains uncertain. Although altered peripheral leukocyte profiles may occur in autoimmune diseases (44–47), whether the lower WBC count observed in this small cluster represents a meaningful immunological feature requires validation in larger cohorts. Meanwhile, Cluster 1 exhibited the lowest frequencies of Tfh and Tfc cells, accompanied by the lowest disease activity. Although the differences in disease duration did not reach statistical significance, patients in Cluster 1 showed a numerical trend toward longer disease duration. This observation raises the possibility that circulating follicular T-cell profiles may be influenced not only by current disease activity but also by disease stage and previous treatment exposure. Although no significant differences in current medication use were observed among clusters, the relatively higher proportion of biological agent use in Cluster 1 may represent one potential factor influencing peripheral immune cell profiles. However, detailed information regarding cumulative treatment duration and longitudinal changes in immune characteristics was unavailable in this study. Furthermore, circulating immune profiles may not fully reflect immune responses occurring within lymphoid tissues or inflamed joints. Therefore, the mechanisms underlying the lower circulating Tfh/Tfc levels observed in Cluster 1 require further investigation through longitudinal studies.

To further delineate the microenvironmental characteristics of these immune subgroups, we analyzed cytokines closely associated with T cell activation. The results revealed that Cluster 3 patients not only had higher disease activity but also higher serum levels of sIL-2R and IL-10 than Cluster 1. As sIL-2R is a circulating marker of T-cell activation, its higher level in Cluster 3 may be compatible with increased systemic T-cell activation (37–39). IL-10 is a pleiotropic cytokine that is widely recognized for its anti-inflammatory properties. However, it appears to exert a pro-proliferative and pro-differentiative effect on B cells under certain circumstances (48, 49). In patients belonging to Cluster 3, the elevated IL-10 levels may reflect complex immune regulation. Given the exploratory nature of the clustering analysis and the small cluster sizes, whether these differences contribute to disease activity or represent secondary immune responses remains uncertain and requires functional and longitudinal investigation.

Notably, the present study is not without limitations. First, this was a single-center study with a relatively small sample size, and no formal *a priori* sample-size calculation was performed before participant recruitment, which may affect the precision of effect estimates, clustering stability, and generalizability of the findings. Secondly, our analyses focused on peripheral blood cells rather than those in affected joints or lymphoid organs. While peripheral blood data reflect systemic immune status, they cannot fully recapitulate the local pathological microenvironment. Finally, as a cross-sectional study, although we observed associations between follicular T-cell-related parameters and clinical characteristics, these findings do not establish causal relationships or clinical predictive value. In the future, larger independent cohorts with longitudinal follow-up are required to determine the reproducibility and clinical relevance of these observations.

## 5 Conclusions

In summary, this study systematically describes the immunological features of Tfh/Tfr/Tfc cells dysregulation in the peripheral blood of RA patients and demonstrates their associations with disease activity, autoantibody levels, and immune activation-related markers. More importantly, exploratory clustering analysis based on Tfh and Tfc profiles identified three distinct follicular T-cell-associated immune patterns with heterogeneous clinical and cytokine characteristics among RA patients. These patterns may provide additional insights into the immunological heterogeneity of RA. However, the biological mechanisms and potential clinical implications of these immune patterns require validation in larger, independent cohorts.

## Data Availability

The raw data supporting the conclusions of this article will be made available by the authors, without undue reservation.
