# Supplementary material for: Immunological dysregulation of follicular helper T cells, follicular regulatory T cells, and follicular cytotoxic T cells is associated with disease activity and identifies exploratory immune patterns in rheumatoid arthritis
Source: Front Immunol. 2026 Sep 4;17:1941650. doi: 10.3389/fimmu.2026.1941650 (PMC13585602; doi:10.3389/fimmu.2026.1941650)
Supplement: Supplementary file 1 [file SupplementaryFile1.pdf]

Supplementary Material

1. Supplementary Methods

1.1 Flow Cytometry Measurement of the Percentages and Absolute Counts of Peripheral Blood $CD4^{+}$T, $CD8^{+}$T, Tfh, Tfr, and Tfc Cell Subsets

1.1.1 Detection of $CD4^{+}$T and $CD8^{+}$T cells

Obtain a BD TrucountTM tube pre-filled with a known quantity of beads. Using a micropipette, dispense 20 μL of the anti-CD3-FITC/CD45-PerCP/CD8-PE/CD4-APC reagent at the base of the tube. Employ the reverse-loading technique to add 50 μL of EDTA-anticoagulated venous blood into the tube. Agitate the contents thoroughly using a vortex mixer and then incubate the tube in a controlled-temperature environment of 20-25 °C for 15-20 minutes, ensuring protection from light. Subsequently, introduce 450 μL of diluted 1X lysing solution and mix vigorously with the vortex mixer. Conduct the red blood cell lysis reaction in an environment maintained at 20-25 °C and shielded from light for 15-20 minutes. Conclude the flow cytometry analysis within 4 hours to establish the absolute count.

1.1.2 Detection of Tfr cells

Utilize a micropipette to transfer 200 μL of fresh peripheral blood sample into a flow-cytometry loading tube. Subsequently, dispense anti-CD4-FITC/CD25-APC/CXCR5-BV421 reagent at the bottom of the tube. After thoroughly mixing the sample with the antibody, incubate the mixture in a controlled-temperature environment of 20-25 °C, shielded from light, for 40 minutes. Next, add 1 mL of fixation/permeabilization solution and homogenize the contents using a vortex mixer. Proceed to maintain the sample in the 20-25 °C temperature - controlled environment, protected from light, for an additional 30 minutes. Then, introduce 2 mL of diluted 1X Buffer and mix well. Allow the sample to stand at 4 °C in the dark for 10 minutes, followed by centrifugation at 1500 revolutions per minute (rpm) for 5 minutes. Discard the supernatant. Add 5 μL of FOXP3-PE antibody marker and mix thoroughly. Incubate the sample in the 20-25 °C temperature-controlled environment, away from light, for 30 minutes. Add 2 mL of diluted 1X Buffer to repeat the washing step once. Finally, add 400 μL of diluted 1X Buffer and prepare the sample for analysis on the flow-cytometry instrument.

1.1.3 Detection of Tfh and Tfc cells

Employ a micropipette to transfer 200 μL of fresh peripheral blood sample into a flow-cytometry loading tube. Subsequently, inject anti-CD4-FITC/CD8-APC-Cy™7/CXCR5-BV421/PD-1-PE reagent at the bottom of the tube. After thoroughly mixing the sample with the antibody, incubate the mixture in a precisely controlled temperature environment ranging from 20-25 °C for 30 minutes, ensuring complete protection from light. Introduce 2 mL of diluted 1X hemolysin solution and homogenize the contents using a vortex mixer. Conduct the erythrocyte lysis reaction within the 20-25 °C temperature range, shielded from light. Subsequently, centrifuge the sample at 1500 revolutions per minute (rpm) for 5 minutes and discard the supernatant. Wash the sample once with 2 mL of phosphate-buffered saline (PBS) buffer. Finally, add 300 μL of PBS buffer and load the sample onto the flow-cytometry instrument for analysis.

1.1.4 Gating strategies and cell counting for flow cytometry analysis

Using the quantitative analysis method of flow cytometry, the relative percentages of Tfh, Tfr, and Tfc cell subsets within $CD4^{+}$T or $CD8^{+}$T lymphocytes were first measured. Subsequently, the percentage of each subset was multiplied by the absolute count value of $CD4^{+}$T or $CD8^{+}$T cells calibrated by the fluorescence

microsphere calibration tube. Ultimately, the absolute counts of the Tfh, Tfr, and Tfc cell subsets were obtained. All the tubes were tested on the flow cytometer (FACSCantoTM II; Becton Dickinson, San Jose, California, USA) within 4 hours. Antibody details are provided in Table S1.

1.2 Detection of Cytokine Concentrations by Cytometric Bead Array

1.2.1 Preparation of reagents and standards

All reagents were equilibrated to room temperature before use. The 10× PBS solution was diluted with distilled water to prepare 0.01 M PBS working solution. The cytokine standards were reconstituted with the supplied diluent according to the manufacturer's instructions and serially diluted to generate standard curves. The standard concentrations were prepared as follows: 2.44, 4.88, 9.77, 19.53, 39.06, 78.13, 156.25, 312.50, 625, 1250, 2500, and 5000 pg/mL.

1.2.2 Sample preparation and incubation

Serum samples were collected from peripheral venous blood and stored at -20 °C until analysis. Before testing, serum samples were thawed and mixed thoroughly. The capture microsphere mixture was prepared according to the manufacturer's instructions and allowed to stand at room temperature for 15–30 min in the dark before use. For each reaction, 25 μL of the capture microsphere mixture containing cytokine-specific antibodies was added to the corresponding assay tube, followed by 25 μL of serum sample, cytokine standard, or blank control.

1.2.3 Detection antibody incubation

Subsequently, 25 μL of PE-conjugated detection antibody mixture was added to each tube. The samples were mixed thoroughly and incubated at room temperature for 2.5–3 h in the dark to allow the formation of antibody–cytokine–microsphere sandwich complexes.

1.2.4 Washing and flow cytometry acquisition

Following incubation, 1 mL of 0.01 M PBS was added to each tube. The samples were centrifuged at 200 × g for 5 min, and the supernatant was carefully removed. The microsphere complexes were resuspended in 100 μL of 0.01 M PBS and analyzed by flow cytometry. Different microsphere populations were identified according to their fluorescence characteristics, and PE fluorescence intensity was recorded for quantitative analysis.

1.2.5 Data analysis and cytokine quantification

Standard curves were generated using recombinant cytokine standards. The concentrations of cytokines in serum samples were calculated based on the corresponding standard curves. The measured cytokines included soluble interleukin-2 receptor (sIL-2R), interleukin-2 (IL-2), IL-4, IL-6, IL-10, IL-17, interferon-γ (IFN-γ), and tumor necrosis factor-α (TNF-α).

2. Supplementary Tables

Table S1	Antibodies used for flow cytometric analysis
Table S2	The summary of demographic data, clinical data and laboratory tests between RA and HC
Table S3	Correlations between circulating follicular T-cell subsets and clinical parameters in patients with RA
Table S4	Clustering analysis of RA patients based on the percentages of Tfh and Tfc cells
Table S5	Summary of demographic characteristics, clinical characteristics, laboratory parameters and treatment information among three exploratory clusters of patients with RA

Table S1 Antibodies used for flow cytometric analysis

Target	Clone	Fluorochrome	Catalogue numbers	Manufacturer
CD3	SK7	FITC	345763	BD Pharmingen™
CD45	2D1	PerCP	345809	BD Pharmingen™
CD4	RPA-T4	APC	555349	BD Pharmingen™
CD8	SK1	PE	555367	BD Pharmingen™
CD4	RPA-T4	FITC	555346	BD Pharmingen™
CD8	SK1	APC-Cy™7	561945	BD Pharmingen™
CD25	M-A251	APC	560987	BD Pharmingen™
CXCR5	RF8B2	BV421	562747	BD Horizon™
CD279(PD-1)	MIH4	PE	560908	BD Pharmingen™
FOXP3	236A/E7	PE	12-4777-42	eBioscience™

Details of the antibodies used for flow cytometric analysis in this study, including the target, clone, fluorochrome, catalogue number, and manufacturer, are provided. All antibodies were used according to the manufacturers' instructions.

Table S2 The summary of demographic data, clinical data and laboratory tests between RA and HC

	HC(N=16)	RA(N=41)	P value
**Demographic information**			
Male, n (%) [c]	6(37.5%)	16(39.0%)	0.915
Female, n (%) [c]	10(62.5%)	25(61.0%)	
Age (years) [b]	60.0(41.50-74.00)	63.00(52.50-68.00)	0.650
Weight(kg) [b]	63.5(60.00-68.00)	62.50(59.50-66.25)	0.516
Height(cm) [a]	159.38±6.56	163.27±7.97	0.088
**Clinical data**			
Disease duration (months) [b]	-	48(12-120)	-
TJC [b]	-	10(3-23)	-
SJC [b]	-	2(0-10)	-
DAS28 [a]	-	5.71±1.56	-
**Laboratory data**			
WBC (×10^9/L) [b]	6.03(4.54-8.44)	6.74(5.61-8.22)	0.404
LY (×10^9/L) [a]	1.70±0.76	1.41±0.53	0.118
Hb (g/L) [a]	136.56±18.44	119.95±15.12	**0.001****
PLT (×10^9/L) [a]	249.25±69.20	268.22±78.24	0.400
ESR (mm/h) [b]	11.50(7.25-23.25)	56.00(16.50-95.50)	**0.001****
CRP (mg/L) [b]	3.00(1.03-9.87)	34.90(8.33-64.21)	**<0.001*****
Liver function			
ALT (U/L) [b]	20.60(14.05-33.93)	18.70(12.50-39.50)	0.709
AST (U/L) [b]	25.75(16.53-28.93)	22.00(15.80-28.35)	0.259
Renal function			
BUN (mmol/L) [b]	5.22(4.33-6.88)	6.30(4.90-7.95)	0.110
Cr (μmol/L) [a]	60.13±14.27	56.73±13.33	0.399
Autoantibody			
Anti-CCP (U/mL) [b]	-	550.00(136.92-550.00)	-
Anti-MCV (U/mL) [b]	-	633.60(66.70,1100.00)	-
RF-IgA (U/mL) [b]	-	73.10(3-237.20)	-
RF-IgG (U/mL) [b]	-	53.40(3-176.60)	-
RF-IgM (U/mL) [b]	-	201.00(35.40-350.00)	-

Abbreviations: HC: healthy control; RA: rheumatoid arthritis; TJC: tenderness joint count; SJC: swollen joint count; DAS 28: disease activity score 28; WBC: white blood cell; LY: lymphocyte; Hb: hemoglobin; PLT: platelet; ESR: erythrocyte sedimentation rate; CRP: C-reactive protein; ALT: alanine transaminase; AST: aspartic transaminase; BUN: blood urea nitrogen; Cr: creatinine; Anti-CCP: anti-cyclic citrullinated peptide; Anti-MCV: anti-mutated citrullinated vimentin; RF: rheumatoid factor.

[a] Results were expressed as the mean±SD and were analyzed by independent-samples t-tests.

[b] Results were expressed as the median (Q1-Q3) and were analyzed by Mann–Whitney U test.

[c] Categorical variables were described as rates and percentages and were assessed using the chi-square tests.

Bold P values indicate statistical significance (P < 0.05). **P<0.01, ***P<0.001.

Table S3 Correlations between circulating follicular T cell subsets and clinical parameters in patients with RA

Table S3A Correlations between follicular T cell subsets and hematological parameters

Immune parameter	Clinical parameter	Spearman rho	95% CI	P value	FDR q value
Tfh%	WBC	-0.041	-0.378-0.310	0.797	0.943
Tfh%	Hb	-0.144	-0.477-0.177	0.368	0.756
Tfh%	PLT	-0.034	-0.353-0.284	0.834	0.943
Tfh%	LY	-0.265	-0.548-0.061	0.094	0.749
Tfc%	WBC	0.084	-0.268-0.437	0.600	0.808
Tfc%	Hb	-0.002	-0.300-0.296	0.990	0.990
Tfc%	PLT	0.134	-0.216-0.468	0.402	0.756
Tfc%	LY	-0.173	-0.478-0.169	0.278	0.756
Tfr%	WBC	-0.281	-0.539-0.008	0.075	0.749
Tfr%	Hb	0.146	-0.155-0.434	0.363	0.756
Tfr%	PLT	-0.128	-0.398-0.164	0.425	0.756
Tfr%	LY	-0.024	-0.335-0.332	0.884	0.943
Tfh/Tfr	WBC	0.148	-0.180-0.458	0.355	0.756
Tfh/Tfr	Hb	-0.188	-0.462-0.116	0.240	0.756
Tfh/Tfr	PLT	0.095	-0.202-0.404	0.553	0.808
Tfh/Tfr	LY	-0.083	-0.396-0.239	0.606	0.808

Table S3B Correlations between follicular T cell subsets and disease activity-related parameters

Immune parameter	Clinical parameter	Spearman rho	95% CI	P value	FDR q value
Tfh%	ESR	0.238	-0.103-0.542	0.134	0.353
Tfh%	CRP	0.055	-0.279-0.378	0.731	0.859
Tfh%	TJC	0.386	0.091-0.627	0.013	0.051
Tfh%	SJC	0.224	-0.107-0.518	0.159	0.353
Tfh%	DAS28	0.434	0.122-0.689	0.005	**0.046***
Tfc%	ESR	0.194	-0.148-0.498	0.223	0.406
Tfc%	CRP	-0.077	-0.409-0.263	0.634	0.849
Tfc%	TJC	0.393	0.111-0.632	0.011	0.051
Tfc%	SJC	0.391	0.079-0.632	0.012	0.051
Tfc%	DAS28	0.503	0.202-0.738	0.001	**0.016***
Tfr%	ESR	0.015	-0.289-0.315	0.927	0.945
Tfr%	CRP	0.011	-0.290-0.305	0.945	0.945
Tfr%	TJC	-0.061	-0.349-0.251	0.705	0.859
Tfr%	SJC	-0.139	-0.418-0.153	0.388	0.646
Tfr%	DAS28	-0.076	-0.363-0.229	0.637	0.849
Tfh/Tfr	ESR	0.081	-0.224-0.367	0.617	0.849
Tfh/Tfr	CRP	-0.032	-0.346-0.294	0.840	0.933
Tfh/Tfr	TJC	0.215	-0.080-0.480	0.177	0.354
Tfh/Tfr	SJC	0.227	-0.099-0.511	0.154	0.353
Tfh/Tfr	DAS28	0.246	-0.060-0.514	0.122	0.353

Table S3C Correlations between follicular T cell subsets and autoantibody profiles

Immune parameter	Clinical parameter	Spearman rho	95% CI	P value	FDR q value
Tfh%	RFIgA	0.107	-0.209-0.417	0.504	0.848
Tfh%	RFIgG	0.055	-0.261-0.355	0.732	0.948
Tfh%	RFIgM	0.100	-0.236-0.405	0.535	0.856
Tfh%	AntiCCP	0.421	0.118-0.672	0.006	**0.048***
Tfh%	AntiMCV	0.415	0.119-0.667	0.007	**0.048***
Tfh%	IgA	-0.031	-0.385-0.333	0.848	0.993
Tfh%	IgG	0.379	0.093-0.613	0.015	0.067
Tfh%	IgM	0.298	-0.025-0.583	0.058	0.187
Tfc%	RFIgA	0.401	0.119-0.646	0.009	**0.050***
Tfc%	RFIgG	0.321	0.010-0.590	0.041	0.162
Tfc%	RFIgM	0.282	-0.010-0.543	0.074	0.214
Tfc%	AntiCCP	0.411	0.143-0.625	0.008	**0.048***
Tfc%	AntiMCV	0.437	0.178-0.643	0.004	**0.048***
Tfc%	IgA	0.024	-0.298-0.331	0.882	0.993
Tfc%	IgG	0.223	-0.112-0.537	0.162	0.392
Tfc%	IgM	0.180	-0.139-0.466	0.261	0.522
Tfr%	RFIgA	-0.004	-0.319-0.315	0.982	0.993
Tfr%	RFIgG	0.119	-0.195-0.406	0.458	0.814
Tfr%	RFIgM	0.146	-0.171-0.449	0.362	0.681
Tfr%	AntiCCP	-0.076	-0.375-0.232	0.637	0.926
Tfr%	AntiMCV	-0.006	-0.307-0.294	0.973	0.993
Tfr%	IgA	0.016	-0.278-0.319	0.920	0.993
Tfr%	IgG	-0.313	-0.572-0.014	0.046	0.165
Tfr%	IgM	-0.078	-0.391-0.230	0.628	0.926
Tfh/Tfr	RFIgA	0.049	-0.252-0.348	0.759	0.948
Tfh/Tfr	RFIgG	-0.059	-0.343-0.239	0.715	0.948
Tfh/Tfr	RFIgM	-0.047	-0.347-0.259	0.770	0.948
Tfh/Tfr	AntiCCP	0.273	-0.033-0.547	0.084	0.224
Tfh/Tfr	AntiMCV	0.213	-0.083-0.485	0.181	0.392
Tfh/Tfr	IgA	0.001	-0.332-0.329	0.993	0.993
Tfh/Tfr	IgG	0.435	0.143-0.667	0.005	**0.048***
Tfh/Tfr	IgM	0.212	-0.132-0.518	0.184	0.392

Abbreviations: Tfh: follicular helper T cells; Tfr: follicular regulatory T cells; Tfc: follicular cytotoxic T cells; WBC: white blood cell; LY: lymphocyte; Hb: hemoglobin; PLT: platelet; ESR: erythrocyte sedimentation rate; CRP: C-reactive protein; TJC: tenderness joint count; SJC: swollen joint count; DAS 28: disease activity score 28; Anti-CCP: anti-cyclic citrullinated peptide; Anti-MCV: anti-mutated citrullinated vimentin; RF: rheumatoid factor; Ig: total serum immunoglobulin. Bold FDR q values indicate statistical significance after FDR correction ($q \leq 0.05$).

Table S4 Clustering analysis of RA patients based on the percentages of Tfh and Tfc cells

	Cluster 1 (n=24)	Cluster 2 (n=3)	Cluster 3 (n=14)	P value
Tfh% [a]	3.56±0.91	10.46±2.06	5.82±1.35	**<0.001*** **
Tfc% [b]	1.10±0.59	6.50±2.12	2.57±1.32	**0.009** **

Abbreviations: Tfh: follicular helper T cells. Tfc: follicular cytotoxic T cells.

[a] Data were normally distributed and showed homogeneity of variance. Continuous variables were expressed as mean ± SD and compared using ANOVA.

[b] Data were normally distributed but showed unequal variances. Continuous variables were expressed as mean ± SD and compared using Welch.

Bold P values indicate statistical significance (P < 0.05). **P<0.01, ***P<0.001.

Table S5 Summary of demographic characteristics, clinical characteristics, laboratory parameters, and treatment information among three exploratory clusters of patients with RA

	Cluster 1(n=24)	Cluster 2(n=3)	Cluster 3(n=14)	P value
**Demographic information**				
Male, n (%) [d]	4(16.7%)	2(66.7%)	10(71.4%)	**0.002****
Female, n (%) [d]	20(83.3%)	1(33.3%)	4(28.6%)	
Age (years) [a]	55.63±11.28	66.00±2.65	67.07±8.01	**0.004****
Weight(kg) [a]	63.71±12.28	63.50±4.82	58.64±9.36	0.393
Height(cm) [a]	162.21±7.36	167.00±6.56	164.29±9.30	0.531
**Clinical data**				
Disease duration (months) [c]	66(30-180)	48(25-84)	24(5-48)	0.117
TJC [c]	5(3-16)	14(7-14)	20(9-24)	**0.040***
SJC [c]	1(0-4)	10(5-12)	10(1-22)	0.055
DAS28 [c]	5.00(4.20-6.30)	4.90(4.55-6.30)	6.90(6.30-7.50)	**0.017***
**Comorbidities**				
Osteoporosis [d]	7(29.2%)	1(33.3%)	6(42.9%)	0.760
Hypertension [d]	8(33.3%)	1(33.3%)	3(21.4%)	0.765
Diabetes [d]	1(4.2%)	0(0%)	4(28.6%)	0.075
Coronary heart disease [d]	1(4.2%)	0(0%)	4(28.6%)	0.075
**Laboratory data**				
WBC (×10^9/L) [a]	7.02±1.80	4.20±1.99	8.02±3.19	**0.049***
LY (×10^9/L) [c]	1.48(1.14-2.00)	1.18(1.00-1.19)	1.22(0.62-1.62)	0.071
Hb (g/L) [a]	118.71±15.66	123.67±9.29	121.29±15.75	0.806
PLT (×10^9/L) [a]	269.13±62.52	268.00±41.33	266.71±107.94	0.996
ESR (mm/h) [a]	54.96±40.51	77.33±62.80	62.14±39.67	0.646
CRP (mg/L) [c]	29.42(8.04-49.35)	20.50(14.37-39.33)	50.57(8.42-85.62)	0.634
Liver function				
ALT (U/L) [c]	16.55(12.50-23.20)	31.80(20.25-49.50)	24.35(16.90-43.30)	0.444
AST (U/L) [c]	20.95(15.50-23.80)	19.90(17.05-25.25)	22.85(17.10-31.50)	0.402
Renal function				
BUN (mmol/L) [b]	5.82±1.64	9.93±4.52	7.16±2.00	0.146
Cr (μmol/L) [a]	54.34±11.82	66.53±22.81	58.71±13.51	0.264
Autoantibody [b]				
Anti-CCP (U/mL) [c]	356.29(36.28-550.00)	550.00(550.00-550.00)	550.00(363.22-550.00)	0.081
Anti-MCV (U/mL) [c]	205.80(16.85-827.85)	1100.00 (735.65-1100.00)	967.10 (331.30-1100.00)	**0.034***
RF-IgA (U/mL) [c]	55.25(3.00-190.05)	26.30(19.85-188.15)	164.15(22.20-350.00)	0.386
RF-IgG (U/mL) [c]	49.30(3.00-154.90)	62.40(40.65-206.20)	65.55(3.00-176.60)	0.580
RF-IgM (U/mL) [c]	155.15(13.25-350.00)	278.50(137.60-314.25)	203.95(38.00-350.00)	0.649
**Current medications**				
Oral prednisone(≤10 mg/day), n (%) [d]	13(54.2%)	2(66.7%)	10(71.4%)	0.617
DMARDs, n (%) [d]	20(83.3%)	3(100.0%)	13(92.9%)	0.754
Biological agent, n (%) [d]	5(20.8%)	0(0%)	1(7.1%)	0.621

Abbreviations: HC: healthy control; RA: rheumatoid arthritis; TJC: tenderness joint count; SJC: swollen joint count; DAS

28: disease activity score 28; WBC: white blood cell; LY: lymphocyte; Hb: hemoglobin; PLT: platelet; ESR: erythrocyte sedimentation rate; CRP: C-reactive protein; ALT: alanine transaminase; AST: aspartic transaminase; BUN: blood urea nitrogen; Cr: creatinine; Anti-CCP: anti-cyclic citrullinated peptide; Anti-MCV: anti-mutated citrullinated vimentin; RF: rheumatoid factor.

[a] Data were normally distributed and showed homogeneity of variance. Continuous variables were expressed as mean ± SD and compared using ANOVA, followed by Bonferroni post hoc correction for pairwise comparisons.

[b] Data were normally distributed but showed unequal variances. Continuous variables were expressed as mean ± SD and compared using Welch.

[c] Data were non-normally distributed. Results were expressed as the median (Q1-Q3) and were analyzed by Kruskal-Wallis H test, followed by Dunn's post hoc test for pairwise comparisons.

[d] Categorical variables were presented as numbers and percentages and compared using Fisher's exact test, followed by Bonferroni post hoc correction for pairwise comparisons.

Bold P values indicate statistical significance ($P < 0.05$). *P<0.05, **P<0.01.

3. Supplementary Figures

Figure S1	A schematic representation of the gating strategy for flow cytometric analysis
Figure S2	The correlation between Tfh, Tfr, and Tfc cells, as well as the Tfh/Tfr ratio, and general clinical indicators in the peripheral blood of RA patients
Figure S3	Internal validation of K-means clustering analysis.

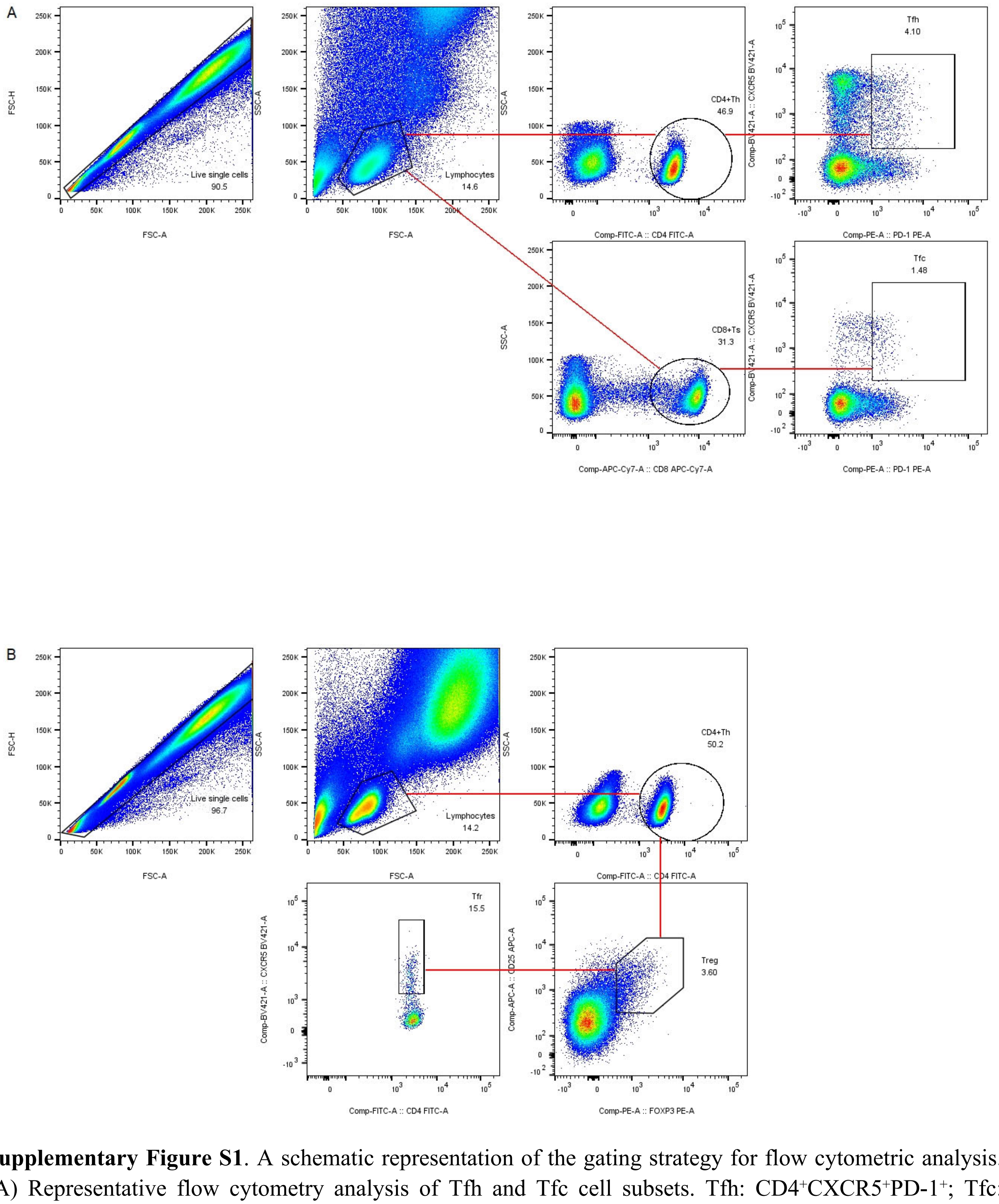

Supplementary Figure S1. A schematic representation of the gating strategy for flow cytometric analysis. (A) Representative flow cytometry analysis of Tfh and Tfc cell subsets. Tfh: $CD4^+CXCR5^+PD\text{-}1^+$; Tfc: $CD8^+CXCR5^+PD\text{-}1^+$. (B) Representative flow cytometry analysis of Tfr cell subsets. Tfr: $CD4^+CD25^+CXCR5^+FOXP3^+$.

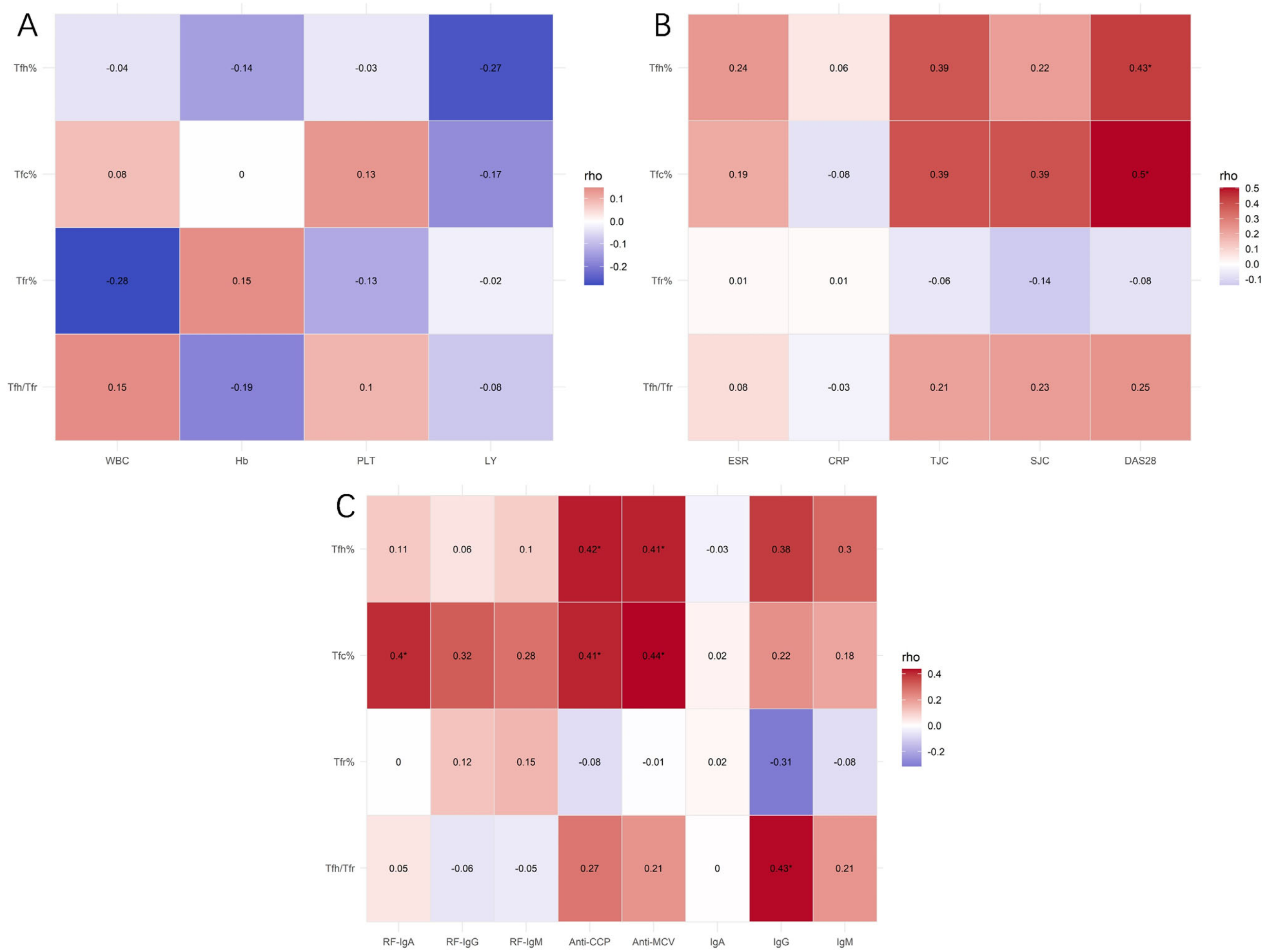

Supplementary Figure S2. Correlation analysis between circulating follicular T-cell subsets and clinical/laboratory parameters in patients with RA. Spearman's rank correlation analysis was performed to evaluate the associations between the percentage of Tfh, Tfr and Tfc, the Tfh/Tfr ratio, and clinical or laboratory parameters in patients with RA. Multiple comparisons were corrected using the Benjamini–Hochberg false discovery rate (FDR) method. Asterisks indicate FDR-adjusted significance levels (*P<0.05). (A) Correlations between follicular T-cell subsets and hematological parameters. (B) Correlations between follicular T-cell subsets and disease activity-related parameters. (C) Correlations between follicular T-cell subsets and autoantibody profiles. Tfh% and Tfr% are presented as percentages of $CD4^{+}$T cells, whereas Tfc% is presented as percentages of $CD8^{+}$T cells. Tfh: follicular helper T cells. Tfr: follicular regulatory T cells. Tfc: follicular cytotoxic T cells. WBC: white blood cell. LY: lymphocyte. Hb: hemoglobin. PLT: platelet. ESR: erythrocyte sedimentation rate. CRP: C-reactive protein. TJC: tenderness joint count. SJC: Swollen joint count. DAS28: disease activity score 28. Anti-CCP: anti-cyclic citrullinated peptide. Anti-MCV: anti-mutated citrullinated vimentin; RF: rheumatoid factor. Ig: total serum immunoglobulin.

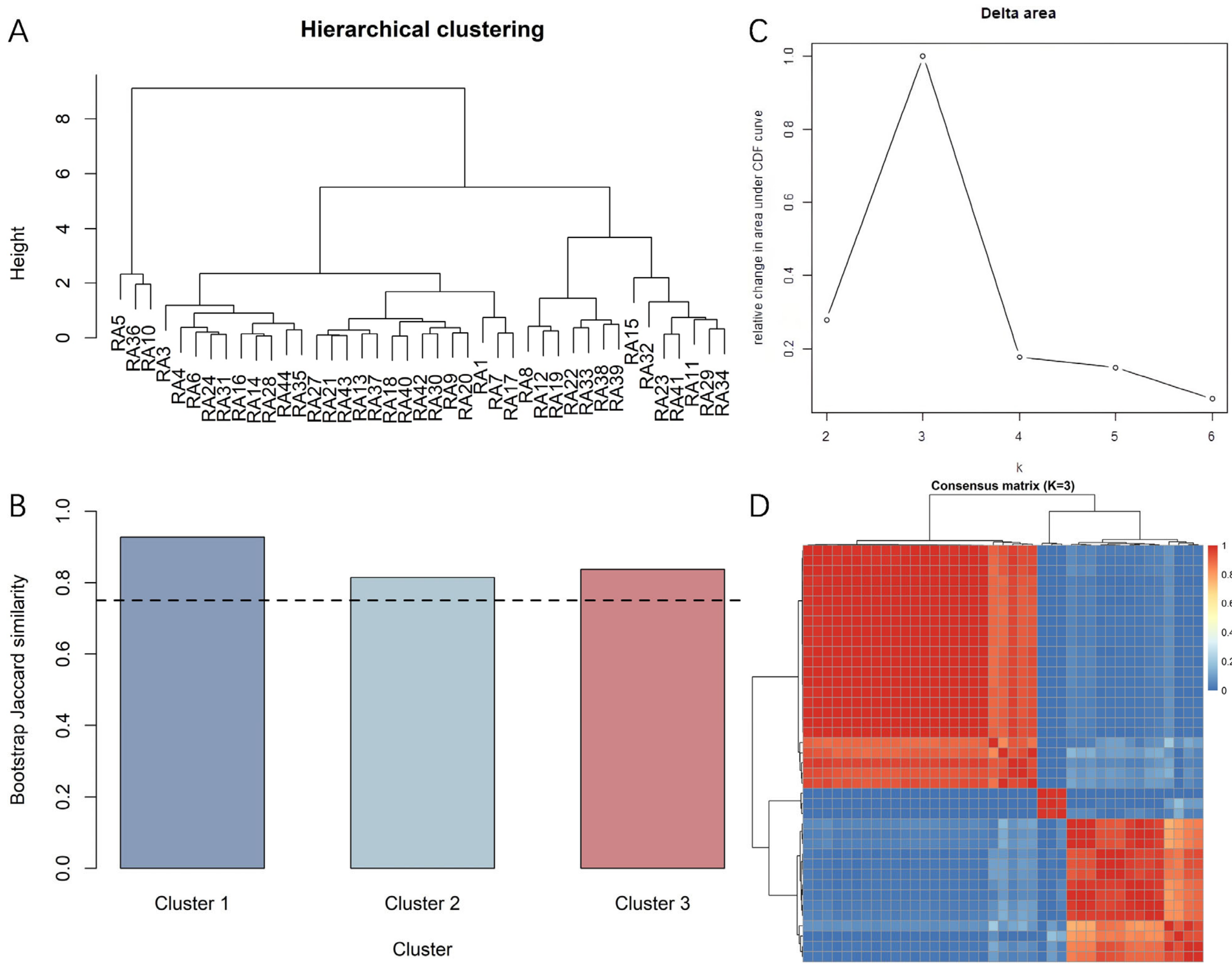

Supplementary Figure S3. Internal validation of K-means clustering analysis. (A) Hierarchical clustering dendrogram based on standardized Tfh% and Tfc% values. The dendrogram demonstrated a three-branch structure. (B) Bootstrap stability analysis showing Jaccard similarity coefficients for each cluster. The mean Jaccard similarity coefficients were 0.9273808 for Cluster 1, 0.8146479 for Cluster 2, and 0.8374362 for Cluster 3. (C) Delta area plot derived from consensus clustering analysis. The relative change in the area under the cumulative distribution function (CDF) curve was evaluated to determine the optimal number of clusters. The largest increase was observed at K=3, supporting the selection of three clusters. (D) Consensus matrix heatmap for the three-cluster solution (K=3). The matrix displays the consensus value between samples, representing the proportion of resampling iterations in which two samples were assigned to the same cluster. Higher consensus values indicate more reproducible co-clustering of samples across iterations, reflecting stronger within-cluster consistency.
